# Visited a vape shop? Prevalence and correlates from a national sample of U.S. young adults

**DOI:** 10.18332/tpc/65203

**Published:** 2016

**Authors:** Shyanika W. Rose, Amy M. Cohn, Jennifer L Pearson, Amanda L Johnson, Jessica M. Rath, Andrea C. Villanti

**Affiliations:** a Schroeder Institute for Tobacco Research and Policy Studies, Truth Initiative, Washington, DC, USA; b Department of Oncology, Cancer Control and Prevention Program, Georgetown University Medical Center, Washington, DC, USA; c Department of Health, Behavior and Society, Johns Hopkins Bloomberg School of Public Health, Baltimore, USA; d Department of Evaluation Science and Research Jobs, Truth Initiative, Washington, DC, USA

**Keywords:** Young adults, Vape shop, Electronic Nicotine Delivery Systems(ENDS)

## Abstract

**INTRODUCTION:**

The increasing popularity of electronic nicotine delivery systems (ENDS) has brought with it the emergence of “vape shops,” retail outlets designed for the sale of ENDS, nicotine fluid, and accessories. To understand user characteristics of this rapidly-growing retail environment, this study examined the prevalence and correlates of having ever visited a vape shop among U.S. young adults.

**METHODS:**

Data were drawn from Wave 7 (weighted n = 3,542) of the Truth Initiative Young Adult Cohort, a national sample of individuals aged 18 to 34 (October 2014). Multivariable logistic analysis examined odds of ever visiting a vape shop as a function of demographics, tobacco use, and substance use.

**RESULTS:**

Results showed that 11% of young adults had ever visited a vape shop. Significant bivariate relationships existed between all tobacco products and other substance use and vape shop visiting. Significant correlates of ever visiting a vape shop in multivariable analyses were: past 30-day use of cigarettes, past 30-day or ever use of an e-cigarette or hookah, past 30-day use of marijuana or other drugs, younger age (18–24 vs 25–34), Hispanic and other race (vs White race), and limited financial means.

**CONCLUSIONS:**

Young adult vape shop ever visitors share common characteristics with tobacco, other drug and ENDS users and likely reflect an underlying propensity to engage in risk behaviors. Future research should determine reasons why young adults visit vape shops and the potential impact of vape shop visits and purchases on ENDS use and trajectories of use of other tobacco products.

## INTRODUCTION

The increasing popularity of electronic nicotine delivery systems (ENDS) has brought with it the emergence of vape shops, retail outlets selling ENDS, nicotine fluid (‘e-liquid’), and related accessories. Vape shops are a distinct retail type that predominantly sells ENDS and e-liquids. Such establishments “sell a variety of products including ENDS, replacement pieces, hardware, custom mixed e-liquids, and other related accessories.”^1^ Hardware sold in vape shops is sometimes known as vaporizer/tank/mod systems (VTMs). Vape shops may allow users to sample a variety of ENDS devices and nicotine fluids, provide advice about choosing devices and nicotine concentration solutions, and sometimes offer “vape lounges” where clients can use ENDS in a social setting^2^. The recently released “Deeming Rule” expanded Food and Drug Administration authority to ENDS products and placed new rules on “ENDS retail establishments” including implementation of 18+ age verification, cessation of free sampling, and inclusion of health warnings on products sold. Vape shops that mix their own e-liquid or customize hardware for direct sale to consumers will be subject to oversight as tobacco product manufacturers.^1^ Prior to FDA authority, there was no standard definition of a vape shop and little is known about how customers view this retail category or who visits these shops.

In 2014, there were an estimated 5,000–10,000 vape shops in the U.S.; generating approximately $500 Min sales^3^. The VTM market is growing at twice the pace of the e-cigarette market, with much of that growth attributed to vape shops^3^, whose sales are expected to rise to $1200M in 2015^4^. Vaporizing devices can also heat marijuana oils/liquid (e.g., “vape pens”)^5^ and it is unknown to what extent vape shop sales reflect purchases for nicotine versus marijuana consumption. A study of students in selected Connecticut high schools found that 5.4% of the total sample and 18% of e-cigarette ever users had used an e-cigarette to consume marijuana^6^.

Since vape shops predominantly sell ENDS, we might expect their customers to be similar to ENDS users. In a 2014 national sample of adults, ever use of e-cigarettes was correlated with daily cigarette smoking, White race, younger age (age 18–24), and living in the Western U.S^7^. One study noted an increase in lifetime e-cigarette use in young adults (aged 18–24) since 2010,^8^ but another showed that ever e-cigarette use remained stable in this age group^9^. A recent national study found that young adults (18–24) were more likely to be some day or every day e-cigarette users than were adults over age 45.^7^ Young adult past 30-day e-cigarette users compared with those with no past-month use have also reported higher prevalence of marijuana use (some days or ever day^10^ or in the past 12 months^11^), some days or every day alcohol use,^10^ past 30-day binge drinking,^11^and past 30-day other tobacco (defined as use of cigars, pipes, chewing tobacco, snuff, snus, hookah, clove cigarettes, bidis, or other) product use^11^. This pattern of e-cigarette co-use with a variety of other substances may indicate a common liability to use addictive substances based on genetic, neurochemical, physiological, behavioral, and/or environmental factors^12–13^. Additionally, over half of U.S. adults (55%) who tried e-cigarettes report that they used them to try to quit smoking cigarettes and e-cigarette users were significantly more likely to quit cigarettes for 24 hours than non-users. However, these relationships may differ among young adults who have not shown a significant relationship between quit intentions and e-cigarette use^14^.

Given the rapid growth in the vape shop market and increased prevalence of trial of ENDS among young adults, there is a need to understand the demographic and substance use characteristics of young adults who visit vape shops. In a prior study of vape shop patrons in the Midwest, participants were primarily White, had attended at least some college, were employed full-time, and had a mean age of 36^15,16^. Using a national sample of U.S. young adults, the purpose of this study was to extend the available research by reporting the prevalence and demographic, tobacco, and substance use correlates of ever visiting a vape shop. Secondly, we describe, associations of vape shop visitation with quitting intentions and behaviors among tobacco users. In line with the common liability model,^12,13^ we would expect that young adult vape shop visitors would be more likely than non-visitors to use e-cigarettes, other tobacco products, and other substances. Findings from this study will inform future research on reasons for visiting vape shops, the impact of federal and state regulation on these establishments, and how visiting vape shops affects health behavior.

## METHODS

### Study Sample

The Truth Initiative Young Adult Cohort Study was designed to understand the trajectories of tobacco use in a young adult population using a longitudinal cohort sample. Details of the cohort have been described elsewhere^17^. The cohort is comprised of a national sample of young adults ages 18–34 drawn from GfK’s KnowledgePanel^®^, an online panel of adults ages 18 and older that covers both the online and offline populations in the U.S. (http://www.gfk.com/products-a-z/knowledgepanelr-north-america/). The panel was recruited via address-based sampling, which accounted for U.S.-based representations by race/ethnicity and cellphone only households. GfK provided internet access and netbooks to households that lacked them to reduce response bias. This methodology has been reported previously,^18,19^ and is used broadly in peer-reviewed literature^20–23^. The baseline survey (Wave 1; n=4,215) was conducted in July 2011, with subsequent assessments occurring approximately every 6 months; the study is ongoing. The cohort is refreshed at each wave to retain the initial sample size. African-American and Hispanic individuals were over sampled at Wave 1 to ensure sufficient sample sizes for subgroup analyses; in Wave 7, younger (aged 18–24) individuals were over sampled in the refresh sample. This study uses data from the Wave 7 survey (n=3,628), collected in October 2014. The panel recruitment rate (RECR) (i.e., percent of individuals consenting to join the panel from those for whom contact was attempted)^24^ for Wave 7 was 13.9%. In 64.4% of the identified households, one member completed a core profile survey in which key demographic information was collected (profile rate – PROR). For this study, only one panel member/household was selected for the study sample and no members outside the panel were recruited. The completion rate (COMR) among eligible panel respondents was 64.4%. The cumulative response rate (CUMRR1) of survey completion among all recruited individuals was 5.4% (the product of RECR, PROR, and COMR). The present analysis focused on a subset of n = 3,542 participants (weighted) who provided information on vape shop visitation. This study was approved by Chesapeake Institutional Review Board, Inc., and online consent was collected from participants before survey self-administration.

#### MEASURES

#### Outcome Measure:

The main outcome measure for this study was respondents’ answers to the question “Have you ever been to a vape shop?” (yes/no). Respondents were not given a definition of a vape shop in the survey.

#### Demographic Factors:

Participants provided information on their: age (grouped 18–24 and 25–34), gender, race/ethnicity (White, non-Hispanic; Black, non-Hispanic; Other, non-Hispanic; and Hispanic) and educational attainment (less than high school, high school, and some college or greater). Subjective financial situation was also assessed as: does not meet basic needs, just meets basic needs, meets needs with a little left, and lives comfortably.

#### Tobacco Use:

Participants were asked separately about ever and past 30-day use of various tobacco products, including cigarettes, large cigars/little cigars/cigarillos (“any cigars”), e-cigarettes, and hookah. One variable capturing mutually exclusive groups of never, ever, and past 30-day use of each tobacco product was created for analyses. In a preamble to the question, e-cigarettes were also extensively defined to include a broad range of ENDS products, including devices that resemble cigarettes (“cigalike”), vape pens, personal vaporizers, and e-hookahs. For descriptive purposes (shown in Table 1), non-daily use was defined as using/smoking the product 1–24 days in the past 30-days and daily use was defined as using/smoking the product 25 or more days in the past 30-days.

#### Substance Use:

Past 30-day use of alcohol, marijuana, and other drugs was determined by asking users of alcohol, marijuana, or other drugs about frequency of use in the past 30-days, with those using >1 days in the past month defined as a past 30-day user. Examples of other drugs listed in the survey included cocaine, heroin, ecstasy, and meth.

#### Quit Attempts and Use of E-cigarettes to Quit Tobacco:

To examine vape shop visitation and quit attempts among ever tobacco users (n=1,853), we also descriptively report if users had ever quit tobacco for 24 hours or ever quit for 3 months and report ever use of e-cigarettes to help quit tobacco among vape shop visitors and among the total tobacco user sample.

### Statistical Analyses

Analyses were conducted using Stata/SE version 14.0, and data were weighted to offset non-response bias. First, prevalence estimates of vape shop visitation were estimated in the young adult sample. Crude logistic regression models were then used to examine associations of vape shop visitation with demographics, tobacco use, and substance use correlates. Next, all correlates that were associated with e-cigarette use in prior studies^9–11^ were entered into a multivariable logistic regression model, controlling for all other variables. Due to small sample sizes, we collapsed daily and non-daily use of tobacco, alcohol, marijuana, and other drugs into past 30-day use in these models.

## RESULTS

### Prevalence of vape shop visiting

The total sample (weighted n = 3,542) included observations from respondents who answered whether they have ever visited a vape shop (Table 1). Respondents were equally male and female, primarily aged 25–34 (64%), and non-Hispanic White (57%). Most had at least some college education (63%), and at least met financial needs with a little left over (63%). The majority reported past 30-day alcohol use (53%), but not marijuana (11%), or other drug use (2%). Fifty-two percent reported ever use of at least one tobacco product and 22% reported past 30-day use of at least one tobacco product.

#### Vape Shop Visitors:

Overall, 11% of respondents had ever visited a vape shop. Vape shop visitors were similar to the total sample in terms of age, gender, race, education, and financial situation. Sixty percent of vape shop visitors reported past 30-day use of any tobacco product compared to 17% of non-visitors (adjusted Wald F-statistic: 121.72; p <0.001) (analyses not shown). Specifically, 38% of vape shop visitors were past 30-day cigarette smokers (vs. 16% in the full sample), 25% were past 30-day e-cigarette users (vs. 5%), 10% were past 30-day any cigar users (vs. 3%), and 7% were past 30-day hookah users (vs. 1%). Conversely, one of the largest differences was that 39% of vape shop visitors had never used e-cigarettes compared to 83% in the full sample. For descriptive purposes in Table 1, we break down past 30-day use into daily and non-daily use. Compared to the full sample, higher percentages of vape shop visitors were daily and non-daily cigarette smokers; non-daily any cigar users; daily and non-daily e-cigarette users; and non-daily hookah users. Daily use of any cigar and hookah did not differ in the vape shop visitor and full samples. Compared to the total sample, a higher percentage of vape shop visitors were past 30-day alcohol (66% vs. 54%), marijuana (30% vs. 11%), or other drug (7%vs. 2%) users.

#### Tobacco users:

Among ever tobacco users, 13% of vape shop visitors reported using an e-cigarette to help quit tobacco compared to 4% of non-visitors (adjusted Wald F-statistic: 28.03; p<0.001).

### Bivariate and Multivariable Logistic Regression Models

Bivariate analyses (Table 2, OR) identified younger age (18–24 vs 25–34), Hispanic race (vs. non-Hispanic White), and lower financial means as correlates of ever visiting a vape shop. Ever or past 30-day tobacco use and past 30-day substance use was associated with higher odds of ever having visited a vape shop. In the multivariable model (Table 2, aOR) adjusting for all covariates, the strongest correlates that emerged of vape shop visitation were ever or past 30-day e-cigarette and hookah use. Past 30-day e-cigarette use was associated with fifteen-fold higher odds of vape shop visitation (aOR15.03, 95% CI: 9.21, 24.53), followed by past 30-day hookah use with six-fold higher odds (aOR6.18, 95% CI: 2.69, 14.23). Those who reported past 30-day use of marijuana (aOR =1.64; 95% CI: 1.07, 2.51),other drugs (aOR =3.41; 95% CI: 1.34, 8.66), or cigarettes (aOR =1.64, 95% CI: 1.01, 2.65) also had significantly higher odds of vape shop visitation. Younger age, Hispanic and other Non-Hispanic race (vs. White), and meeting basic financial needs with a little left (vs. “live comfortably”) were also positively associated with vape shop visitation. Gender, education, past 30-day alcohol use, and ever use of cigarettes or any cigar use were not significantly correlated with vape shop visitation in the adjusted model.

## DISCUSSION

Our study shows that in 2014, approximately one in 10 young adults nation wide had ever visited a vape shop, compared to other research showing that approximately 1 in 4 young adults have ever visited a hookah bar/lounge^17^. As sales in this retail venue increase nationwide,^4^ we expect the number of young adult visitors to grow. In bivariate models, all tobacco and substance use variables were associated with vape shop visiting. In a multivariable model, perhaps unsurprisingly, past 30-day ENDS users had the highest odds of ever having visited a vape shop and ever ENDS users were also significantly more likely to have ever visited a vape shop. Almost 40% of ever vape shop visitors also reported never using an e-cigarette (which were broadly defined in the survey to include all ENDS) suggesting that vape shop visitors may have visited for other reasons than ENDS use (e.g., socializing, browsing) or ENDS purchase (e.g., purchasing other products).

Past 30-day cigarette users were also more likely to visit a vape shop, suggesting visiting by smokers. Additionally, among ever tobacco users, vape shop visitors were more likely than other tobacco users to report having ever used an e-cigarette to help quit tobacco, which is in line with an earlier study, which showed that adult vape shop customers reported starting ENDS as a means of smoking cessation^16^. However, in this sample, ever vape shop visitors were no more likely to report having ever quit for 24 hours or 3 months than were non-visitors.

Past 30-day or ever use of hookah was also significantly associated with vape shop visitation. It is possible that hookah users may also obtain e-hookah products from vape shops or that such stores could sell both hookah and ENDS supplies^25^. With regard to other substance use, past 30-day other drug and marijuana use were both associated with higher odds of vape shop visits, conforming to the common liability model^13–26^. These findings may highlight a role for vape shops in intervention with multiple tobacco product use (poly-use) or poly-substance use, as previously has been done for smoking cessation^16–27^. Some vape shops also could be selling products for use in the emerging legal marijuana market^28^. However, past 30-day cigarette use was attenuated, and ever cigarette and any use of cigars were not associated with lifetime vape store patronage in adjusted models. This may be because most individuals using hookah and ENDS (or cigars) in this sample were also smoking cigarettes, reflecting dual or poly-tobacco product use and reducing the variance for single products alone.

Demographic characteristics of vape shop visitors in this study are largely but not wholly consistent with characteristics of ENDS users found in prior national studies. For example, among adults, prior studies have found lower odds of past 30-day ENDS use among Hispanics compared with non-Hispanic whites and no difference in past 30-day e-cigarettes use by income^9^, but this study found higher odds of vape shop visiting among Hispanics and non-Hispanics of other races and among respondents who met their needs with a little left compared to those who live comfortably. This is, however, consistent with Hispanic high school students reporting e-cigarettes as the most used product in 2014^29^.

This study has several strengths. First, this study uses an existing online panel to recruit a large, national cohort of young adults. National data show that young adults are more likely to try e-cigarettes than older adults^7^. Determining the characteristics of young adults who visit vape shops is of great interest in understanding the possible role of vape shops in broader patterns of tobacco use. Secondly, this is the first national study to examine characteristics of vape shop visitors among young adults -a high-risk population for tobacco and other substance use. Other groups, such as older adults, may have different characteristics. In future waves of this study, we will be able to detect changes in prevalence of vape shop visitation in this young adult sample and the predictors of such changes.

There are several limitations of this study. First, this is a cross-sectional study and thus temporality cannot be determined and causality cannot be inferred. We cannot conclude whether measured substance use factors promote vape shop visiting, or the converse, whether visiting a vape shop leads to higher risk behaviors, or whether both substance use behaviors and vape shop visiting co-occur as part of an underlying propensity toward engaging in a variety of health-risk behaviors. Secondly, low response to the panel must be considered when generalizing the study findings to the young adult population. Previous estimates from this cohort for key outcomes of interest, such as ever and past 30-day cigarette use, are consistent with other national survey data^17^. However, tobacco use prevalence varies by the way the question is asked^30^ and it is possible that asking about all products in a single list rather than as individual items underestimated the prevalence of use of tobacco products, including e-cigarettes. Thirdly, some factors associated with vape shop visiting may be due to geographic factors of vape shop accessibility, density, and marketing, which were not measured in the current study. Still, the correlates of vape shop visiting were robust across a wide range of covariates previously associated with e-cigarette use. There is no current national sampling frame or comprehensive surveillance of vape shops in the U.S.,^31^ so future research will be necessary to disentangle vape shop visiting behavior from access to and density of these outlets. Fourthly, it is important to note that respondents in this sample were not provided with a definition of a vape shop, so may include visits to stores that sell ENDS along with cigarettes, hookah, other tobacco products, or marijuana-related products. Finally, since vape shops are a relatively new feature of the retail landscape, we focused on whether respondents had ever visited a vape shop in their lifetime and not on what they did while there (purchased a product, vaped or neither). Future research, planned in future waves of this ongoing longitudinal study, will be needed to distinguish “regular” vape shop visitors from those who may have visited only once and if or how vape shop visitation is related to future ENDS uptake or use patterns and related tobacco use trajectories either into or out of use of more or less harmful lifetime exposures.

## CONCLUSION

This study of U.S. young adults demonstrates that vape shop visiting is relatively uncommon (11%) in the general population of young adults. For comparison, vape shop visitation is lower than the 23% of young adults who ever visited a hookah bar, published from an earlier wave of this cohort^17^. Future studies will examine whether vape shop visitation increases if these venues become more prevalent and established in the U.S. Currently, young adult vape shop visitors appear to share some characteristics with ENDS users, including a higher prevalence of tobacco and substance use^10–11^. Consistent with a common liability model, vape shop visitors are using ENDS, other tobacco products, and other substances. Vape shops may have a positive effect on public health if these venues provide cessation support and advice for smokers who are otherwise unable to quit^27–32^. However, these venues may have a negative impact on public health if they facilitate never tobacco users, especially youth, who may not otherwise have used nicotine products, to start using e-cigarettes and progressing to longer-term combustible tobacco use^32^. Among current smokers of combustible tobacco products, the possible harms would also outweigh the benefits if vape shop access promoted ENDS use that slowed, rather than accelerated, population rates of smoking cessation beyond what would have occurred in the absence of vape shops. Currently, the role of these stores as potentially positive, negative, or neutral for public health is unknown.

As the Deeming Rule places new restrictions on vape shops in the U.S., this paper provides valuable information on young adult vape shop visitors to assess how future changes in visiting or characteristics of visitors of these establishments may change over time. Future research is also needed to determine reasons why young adults visit vape shops, their experiences in these stores, their purchases and other behaviors in vape shops, and their subsequent ENDS and tobacco use trajectories to understand the potential impact of this rapidly emerging retail environment on health behavior.

## Figures and Tables

**Table 1:** Characteristics of U.S. young adult vape shop visitors; Truth Initiative Young Adult Cohort, October 2014 (weighted N=3,542)

	Total Sample (N=3,542)		Vape Shop Visitors (n=399)	
	*Total (%)*	*95% CI (%)*	*Yes (%)*	*95% CI (%)*
**Ever visited a vape shop**	-	-	11.3	(10.10, 12.56)
**Age**				
**18–24**	36.4	(34.55, 38.19)	43.4	(37.87, 49.14)
**25–34**	63.7	(61.81, 65.45)	56.6	(50.86, 62.13)
**Sex**				
**Male**	49.6	(47.70, 51.57)	54.6	(48.86, 60.11)
**Female**	50.4	(48.43, 52.30)	45.5	(39.89, 51.14)
**Race**				
**White, non-Hispanic**	57.0	(55.01, 58.98)	53.5	(47.63, 59.29)
**Black, non-Hispanic**	13.3	(11.81, 15.01)	9.3	(6.02, 14.22)
**Other**	8.6	(7.52, 9.89)	11.0	(7.62, 15.62)
**Hispanic**	21.0	(19.42, 22.75)	26.2	(21.17, 31.83)
**Education completed**				
**Less than high school**	11.3	(9.71, 13.09)	14.5	(9.77, 20.85)
**High school**	25.4	(23.66, 27.21)	27.6	(22.82, 33.02)
**At least some college**	63.3	(61.27, 65.32)	57.9	(51.88, 63.73)
**Subjective Financial Situation** a				
**Don’t meet basic expenses**	7.8	(6.68, 9.02)	9.1	(6.19, 13.03)
**Just meet basic expenses**	29.3	(27.49, 31.13)	30.8	(25.58, 36.52)
**Meet needs with a little left**	39.4	(37.56, 41.29)	43.5	(37.98, 49.24)
**Live comfortably**	23.6	(21.98, 25.19)	16.7	(12.60, 21.67)
**Cigarette use**				
**Never**	57.6	(55.71, 59.53)	28.6	(23.59, 34.25)
**Ever**	26.5	(24.90, 28.17)	33.5	(28.44, 38.86)
**Non-daily** d	6.2	(5.31, 7.21)	13.8	(10.44, 17.93)
**Daily** e	9.7	(8.49, 11.01)	24.2	(19.24, 29.87)
**Any cigar use** b				
**Never**	67.1	(65.20, 68.84)	37.62	(32.30, 43.26)
**Ever**	29.6	(27.84, 31.37)	52.59	(46.81, 58.30)
**Non-daily** d	3.0	(2.36, 3.70)	9.01	(5.95, 13.43)
**Daily** e	0.4	(0.23, 0.78)	0.77	(0.23, 2.53)
**E-cigarette use**				
**Never**	82.8	(81.33, 84.22)	39.3	(33.79, 44.98)
**Ever**	12.2	(10.95, 13.47)	35.3	(29.80, 41.13)
**Non-daily** d	3.7	3.06, 4.48)	15.5	(11.87, 19.86)
**Daily** e	1.3	(0.97, 4.48)	10.0	(7.23, 13.81)
**Hookah use**				
**Never**	81.3	(79.90, 82.68)	53.6	(47.83, 59.27)
**Ever**	17.3	(15.96, 18.62)	39.4	(34.01, 45.04)
**Non-daily** d	1.3	(0.93, 1.88)	6.8	(4.16, 11.03)
**Daily** e	0.1	(0.026, 0.39)	0.2	(0.026, 1.29)
**Alcohol use (past 30days)**				
**No**	46.5	(44.58, 48.46)	34.5	(28.88, 40.27)
**Yes**	53.5	(51.54, 55.42)	65.7	(59.73, 71.12)
**Marijuana use (past 30days)**				
**No**	88.7	(87.24, 89.92)	70.5	(64.72, 75.64)
**Yes**	11.4	(10.08, 12.76)	29.5	(24.36, 35.28)
**Other drug use (past 30days)**				
**No**	98.5	(97.86, 98.90)	92.8	(88.79, 95.42)
**Yes**	1.5	(1.10, 2.14)	7.2	(4.58, 11.21)
**Ever quit tobacco for 24 hours** c				
**No**	48.5	(45.83, 51.23)	43.2	(37.04, 49.62)
**Yes**	51.5	(48.77, 54.17)	56.8	(50.38, 62.96)
**Ever quit tobacco for 3 months** c				
**No**	65.3	(62.60, 67.88)	62.5	(56.30, 68.42)
**Yes**	34.7	(32.12, 37.40)	37.5	(31.58, 43.70)
**Ever used e-cigarettes to help quit tobacco** c				
**No**	96.0	(94.85, 96.90)	86.7	(82.07, 90.30)
**Yes**	4.0	(3.10, 5.15)	13.3	(9.70, 17.93)

a n= 9 (weighted) observations missing subjective financial situation. No individuals with missing financial situation reported vape shop visitation.

b Large cigars and little cigars/cigarillos (Any Cigars)

c Among ever tobacco users (weighted n=1853)

d Non-daily = used/smoked 1–24 days in the past 30-days

e Daily = used/smoked 25 or more days in the past 30-days

**Table 2.** Crude odds ratios (OR) and adjusted odds ratios (aOR) of correlates of ever having visited a vape shop among U.S. young adults; Truth Initiative Young Adult Cohort, October 2014 (n =3,533^a^)

Table 2. Correlates of ever having visited a vape shop, among the full weighted sample, wave 7				
	*Bivariate Results OR*	*95% CI*	*Multivariable Results aOR*	*95% CI*
**Age**				
**18–24**	1.40	(1.09, 1.78)	1.68	(1.26, 2.24)
**25–34**	Referent		Referent	
**Gender**				
**Female**	Referent		Referent	
**Male**	1.25	(0.98, 1.59)	1.15	(0.86, 1.54)
**Race**				
**White, non-Hispanic**	Referent		Referent	
**Black, non-Hispanic**	0.72	(0.44, 1.20)	1.26	(0.72, 2.21)
**Other, non-Hispanic**	1.42	(0.91, 2.20)	2.08	(1.26, 3.42)
**Hispanic**	1.38	(1.02, 1.86)	1.73	(1.22, 2.46)
**Education**				
**Less than high school**	0.83	(0.49, 1.39)	1.09	(0.61, 1.95)
**High school diploma/GED**	0.68	(0.42, 1.11)	1.06	(0.75, 1.51)
**At least some college**	Referent		Referent	
**Financial Situation**				
**Don’t meet basic expenses**	1.75	(1.03, 2.96)	1.07	(0.51, 2.28)
**Just meet basic expenses**	1.55	(1.05, 2.29)	1.28	(0.84, 1.95)
**Meet needs with a little left**	1.64	(1.15, 2.35)	1.61	(1.11, 2.33)
**Live comfortably**	Referent		Referent	
**Cigarette use**				
**Never**	Referent		Referent	
**Ever**	2.80	(2.06, 3.79)	1.51	(0.99, 2.31)
**Past 30-day**	6.22	(4.49, 8.62)	1.64	(1.01, 2.65)
**Any cigar use** c				
**Never**	Referent		Referent	
**Ever**	3.71	(2.87, 4.81)	1.23	(0.83, 1.81)
**Past 30-day**	7.17	(4.29, 11.98)	1.44	(0.68, 3.03)
**Hookah use**				
**Never**	Referent		Referent	
**Ever**	4.32	(3.34, 5.59)	2.15	(1.54, 3.00)
**Past 30-day**	15.63	(7.80, 31.30)	6.18	(2.69, 14.23)
**E-cigarette use**				
**Never**	Referent		Referent	
**Ever**	8.61	(6.33, 11.72)	4.90	(3.33, 7.21)
**Past 30-day**	23.69	(16.15, 34.76)	15.03	(9.21, 24.53)
**Alcohol use (past 30-day)**				
**No**	Referent		Referent	
**Yes**	1.77	(1.35, 2.31)	0.81	(0.57, 1.15)
**Marijuana use (past 30-day)**				
**No**	Referent		Referent	
**Yes**	4.21	(3.10, 5.73)	1.64	(1.07, 2.51)
**Other drug use (past 30-day)**				
**No**	Referent		Referent	
**Yes**	9.46	(4.79, 18.69)	3.41	(1.34, 8.66)

a n= 9(weighted) were excluded from the adjusted model due to missing financial situation.

Items in bold indicate a significant association.

c Large cigars and/or little cigars/cigarillos (‘Any Cigar’)
